# A Pilot Study Assessing the Effects of Goal Management Training on Cognitive Functions among Individuals with Major Depressive Disorder and the Effect of Post-Traumatic Symptoms on Response to Intervention

**DOI:** 10.3390/brainsci12070864

**Published:** 2022-06-30

**Authors:** Jenna E. Boyd, Brahm D. Sanger, Duncan H. Cameron, Alina Protopopescu, Randi E. McCabe, Charlene O’Connor, Ruth A. Lanius, Margaret C. McKinnon

**Affiliations:** 1 Department of Psychiatry and Behavioural Neurosciences, McMaster University, Hamilton, ON L8S 3L8, Canada; boydj@stjoes.ca (J.E.B.); dhendersoncameron@ryerson.ca (D.H.C.); rmccabe@stjosham.on.ca (R.E.M.); 2 Anxiety Treatment and Research Clinic, St. Joseph’s Healthcare Hamilton, Hamilton, ON L9C 0E3, Canada; 3 Department of Psychology, Neuroscience and Behaviour, McMaster University, Hamilton, ON L8S 3L8, Canada; sangerb@mcmaster.ca (B.D.S.); protopa@mcmaster.ca (A.P.); 4 Homewood Health Centre, Guelph, ON N1E 6K9, Canada; coconnor@homewoodhealth.com; 5 Department of Psychiatry, Western University, London, ON N6C 5J1, Canada; ruth.lanius@lhsc.on.ca; 6 Lawson Health Research Institute, London, ON N6C 2R5, Canada; 7 Homewood Research Institute, Guelph, ON N1E 6K9, Canada; 8 Mood Disorders Program, St. Joseph’s Healthcare Hamilton, Hamilton, ON L9C 0E3, Canada

**Keywords:** cognitive dysfunction, cognitive remediation, goal management training, major depressive disorder, post-traumatic stress disorder

## Abstract

Recent meta-analyses highlight alterations in cognitive functioning among individuals with major depressive disorder (MDD), with performance deficits observed across multiple cognitive domains including executive functioning, memory, and attention. Moreover, impaired concentration is a formal diagnostic criterion for a major depressive episode. Notably, cognitive impairment is reported frequently in MDD and is associated with poor treatment response. Despite this knowledge, research examining the effectiveness of top-down, adjunctive treatments for cognitive dysfunction in MDD remains in its infancy. The primary aim of the present study was to perform a pilot investigation of the implementation of a standardized cognitive remediation program, Goal Management Training (GMT), among individuals with a primary diagnosis of MDD. A secondary aim was to explore how comorbid symptoms of post-traumatic stress disorder (PTSD) among those MDD patients exposed to trauma may affect treatment response. A final sample of thirty individuals were randomized to either participate in the nine-week GMT program (active group; *n* = 16) or to complete a nine-week waiting period (waitlist control; *n* = 14). One participant was excluded from the GMT group analysis following study completion due to meeting an exclusion criteria. In total, 60% of the individuals allocated to the GMT program were trauma exposed (*n* = 9). Groups were assessed at baseline, post-treatment, and at three-month follow-up. The assessment comprised neuropsychological tasks assessing a variety of cognitive domains, subjective measures of functioning and symptom severity, as well as a clinical interview to establish a primary diagnosis of MDD. Significant gains in processing speed, attention/concentration, and response inhibition were observed for the participants in the GMT condition relative to participants in the waitlist control condition. Individuals in the GMT condition also reported improvements in subjective cognitive functioning from baseline to post-treatment. Heightened PTSD symptom severity was associated with reduced response to treatment with respect to the domain of processing speed. The results of this pilot investigation highlight not only the potential utility of GMT as an augmentative treatment in MDD, but also highlight the contribution of comorbid symptoms of PTSD to diminished treatment response among trauma-exposed individuals with MDD. The study is limited primarily by its small pilot sample and the absence of a program evaluation component to gauge participant opinions and feedback of the treatment protocol.

## 1. Introduction

With a lifetime prevalence of 16.6% in North America [1], major depressive disorder (MDD) is a common and chronic disorder characterized primarily by sustained periods of low mood and a lack of interest in activities one normally enjoys [2]. In addition, MDD is associated with a reduction in the ability to experience positive affect, such as happiness or joy. Notably, MDD may develop following exposure to traumatic events and is commonly comorbid with post-traumatic stress disorder (PTSD) [3,4,5]. For example, 30–50% of individuals with PTSD have also been found to meet diagnostic criteria for a depressive disorder [3]. In addition to common affective and physical symptoms, both disorders are associated with notable cognitive difficulties, with difficulty concentrating among the formal symptoms described for each in the Diagnostic and Statistical Manual for Mental Health Disorders-5-Text Revision (DSM-5-TR) [2]. Indeed, meta-analyses of cognitive functioning in MDD point towards small to moderate effect sizes for deficits across a range of cognitive domains, most notably in executive functioning (Cohen’s d = −0.52 to −0.61) and attention (d = −0.22 to −0.54) [6]. Similarly, meta-analyses of cognition in PTSD also reveal moderate effect size impairment across several cognitive domains, including executive functioning (d = −0.45), verbal learning (d = −0.63) and memory (d = −0.46), as well as visual learning (d = −0.27) and memory (d = −0.32) [7].

Critically, although there has been some debate about whether cognitive impairment is a state or a trait characteristic in MDD, patients with MDD in full remission tend to exhibit worse performance in cognitive domains, such as executive functioning and processing speed relative to healthy controls; although this pattern of findings may be most strongly associated with late-onset depression [8]. Moreover, as in depression, cognitive difficulties in PTSD frequently persist following treatment (e.g., cognitive processing therapy and prolonged exposure) [9]. Trauma exposure in MDD has further implications for patient outcomes; heightened dissociative symptoms among trauma-exposed individuals with MDD are associated with diminished processing speed and reductions in visuospatial recall and verbal recognition [10]. 

Importantly, cognitive impairment among these neuropsychiatric conditions has been associated with reduced functional outcomes [11,12], diminished response to pharmacological treatment [13], and attenuated response to behavioral treatment [14,15]. Despite these well-documented deficits in cognitive function and their potential impact on functional and treatment outcomes, research examining cognitive remediation approaches that directly target cognitive function in MDD remains limited. The extant research, however, points towards generally positive effects of cognitive remediation approaches in MDD. For example, a recent meta-analysis of interventions aimed at targeting cognitive performance in MDD found moderate effects of computerized cognitive training on attention and working memory, as well as improvements in symptom severity, daily functioning, and global functioning [16].

Previous studies surrounding cognitive remediation in MDD have generally investigated bottom-up approaches (i.e., those targeting more basic cognitive abilities, such as attention), as implemented in many computerized cognitive training protocols that rely on drill and practice learning across limited cognitive domains, such as working memory and attention (e.g., [17,18,19]). By contrast, only a limited number of studies have explored top-down protocols aimed at introducing compensatory strategies for improving cognitive functioning among individuals with MDD, which often combine cognitive therapy alongside a memory support intervention. For example, a novel memory support program in addition to cognitive therapy for adults with MDD was associated with documented improvements in recall of therapy sessions relative to those who received treatment as usual [20] (see also [21]). Additional research has also examined combining bottom-up and top-down approaches, which also demonstrates some promise [22]. Specifically, therapist-led, computerized, drill-based exercises in conjunction with metacognitive skills training applied to daily living led to improvements in attention and verbal memory in individuals with treatment-resistant depression in addition to functional improvement [22]. Thus, although early work investigating top-down approaches to cognitive remediation in MDD is promising, the relative dearth of studies in this area highlights an important gap in the field.

Goal Management Training (GMT) is an established top-down cognitive remediation program aimed at improving executive functioning via teaching participants a sequential series of skills to monitor and improve goal-directed behaviors [23,24]. This program utilizes a top-down approach by focusing on higher-order cognitive functions through the introduction of psychoeducation, mindfulness, and other skills, such as goal setting and self-monitoring, with the aim of improving supervisory control [23,24]. The program is typically presented in a group format in order to foster group-specific benefits, such as those seen in group cognitive behavioral therapy (CBT) [25], including sharing and normalization of experience, as well as accountability. GMT has been administered successfully in a variety of clinical and non-clinical populations, such as those with acquired brain injury, substance abuse, spina bifida, as well as older adults, where a recent meta-analysis highlights consistent small-to-medium effects of improvements across a range of cognitive domains, particularly executive function (Hedges’ g = 0.23 to 0.55) and long-term memory (Hedges’ g = 0.27) [26].

GMT has also begun to be applied to other neuropsychiatric conditions, including MDD. For example, Hagen et al. (2020) evaluated the effectiveness of GMT in a randomized controlled trial in MDD using the Norwegian-language adaptation of GMT [27]. At six-month follow-up, GMT was associated with significant improvement on self-report measures of executive functioning and depressive symptoms. GMT has also been associated with improvements in executive functioning, processing speed, verbal memory, and symptom severity in individuals with PTSD [28,29], and in executive functioning and subjective cognition in individuals with obsessive-compulsive disorder [30]. GMT may also impact patterns of emotional response, improving performance on self-report and objective measures of emotion regulation and impulsivity [28,29,30]. Notably, in these studies, GMT appears to slow responding on challenging cognitive tasks, including when under emotional distress (i.e., stopping to think before acting impulsively, a skill vital to emotion regulation, including the management of anger and inappropriate comments and actions).

Due to the promising effects of this top-down approach in multiple populations, the primary aim of the present study was to apply GMT in individuals with MDD. Specifically, our primary aim was to examine the efficacy of GMT among an English-speaking sample of individuals with MDD seeking treatment in a tertiary care setting (i.e., to determine whether this protocol results in significant improvements in cognitive measures, such as executive functioning, attention and memory, subjective cognition, and measures of psychological well-being, such as symptom severity compared to a wait list control (WLC) group). We hypothesized that, relative to the WLC group, participants in the GMT group would should show greater improvements on outcome measures, particularly on measures of executive function. A secondary aim of the study was to explore the influence of comorbid PTSD symptom severity among trauma-exposed individuals with MDD, where PTSD is further associated with cognitive impairment and may reduce response to treatment. Accordingly, we explored the relation between severity of PTSD symptoms and response to treatment (i.e., GMT) among a subset of trauma-exposed individuals with MDD in the GMT group. We hypothesized a decreased response to treatment amongst individuals with greater severity of PTSD symptoms. A final objective was to identify outcome measures of interest using exploratory analyses for future large-scale investigations of GMT in neuropsychiatric populations.

## 2. Materials and Methods

### 2.1. Participants

**Figure 1:**
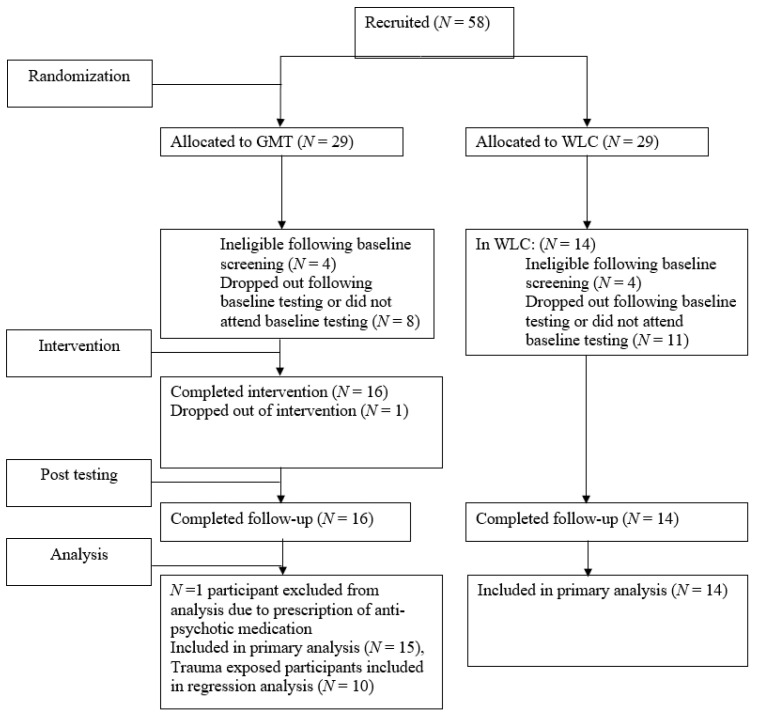
GMT, Goal Management Training; WLC, Waitlist Control.

Fifty-eight (*n* = 58) participants with a principal diagnosis of MDD according to DSM-IV-TR or DSM-5 [2,31] were recruited from the outpatient Mood Disorders Program at St. Joseph’s Healthcare Hamilton and invited to participate in this study. The inclusion criteria were: (1) a principle diagnosis of major depressive disorder confirmed by the Mini International Neuropsychiatric Interview 6.0 or 7.0 (M.I.N.I.) [32]; (2) between the ages of 18 and 65; and (3) able to provide written and informed consent. Participants were excluded if they: (1) were receiving treatment with anti-cholinergic or anti-psychotic medication known to adversely affect cognition; (2) had undergone ECT within the past year; (3) had a history of substance dependence or significant and recent (<1 year) substance abuse; (4) had a recent history (within the past 12 months) of a medical disorder known to adversely affect cognition; (5) loss of consciousness greater than 1 min or a history of traumatic brain injury; or (6) a learning disorder or other disorder known to adversely affect cognition such as a diagnosis of attention-deficit/hyperactivity disorder (ADHD). Participants were not required to discontinue or modify other treatments they were receiving concurrently for MDD (e.g., antidepressant medication, psychotherapy). Twenty-nine *(n* = 29) individuals dropped out or were excluded from the study between baseline (time 1) and post-treatment testing (time 2). Eight (*n* = 8) individuals who were recruited to the study and randomized were deemed to meet exclusion criteria during the first testing session. Within the GMT group, eight individuals did not attend baseline testing after being screened or dropped out prior to the first group GMT session, and one individual dropped out after the first group GMT session. One GMT participant was excluded from analysis following group completion due to use of anti-psychotic medication known to adversely affect cognition. Within the WLC group, 11 individuals did not attend baseline testing or dropped out after baseline testing. Thus, a final sample of twenty-nine *(n* = 29) individuals were included in analysis following participation in the study, across both conditions. Among the final sample within the GMT group, nine participants *(n* = 9) met criterion A for trauma exposure on the Clinician-Administered PTSD Scale for DSM-5 (CAPS-5) [33] and were included in analyses of PTSD severity and response to treatment. See Figure 1 for a chart depicting study recruitment, allocation, and loss to follow up.

### 2.2. Experimental Design and Procedure

Participants were assigned randomly (using https://www.sealedenvelope.com/simple-randomiser/v1/lists) to participate in either GMT or a 9-week waitlist control condition. Participants were assessed at baseline, post-treatment, and 3-month follow-up. The experimental design is a 2 (group) × 3 (time) repeated-measures factorial design. Participants randomized to the waitlist control condition were informed that they would have the opportunity to participate in a therapist-led GMT group treatment at the end of the study.

Participants who had provided consent to be contacted regarding participation in research studies upon referral to the Mood Disorders Outpatient Clinic were contacted by trained study personnel (e.g., graduate and undergraduate students) to review the study in detail and if interested, were invited to participate in the study. Participants were also recruited via self-referral generated through advertisements on public forums (e.g., kijiji.ca and posters) or were referred to the study by their Mood Disorder Outpatient clinicians (e.g., psychiatrists, nursing staff, and social workers). A detailed informed consent form was reviewed and signed at the initial study appointment. At study entry, participants completed a battery of self-report symptom and subjective cognition measures. Participants also completed neuropsychological testing to assess executive functioning, attention, and memory (see below), as well as several functional outcome measures. All outcome measures were completed/administered at baseline (time 1), post-treatment (time 2) and at 3-month follow-up (time 3). Participants completed a structured diagnostic assessment, the M.I.N.I. 6.0 or 7.0 [32] and the CAPS-5 [33] at baseline. Trained researchers at the graduate level or higher administered all study measures under the supervision of a clinical psychologist.

### 2.3. Study Conditions

#### 2.3.1. GMT

GMT is a structured, short-term, cognitive remediation program with an emphasis on mindfulness and practice in planning and goal-oriented behaviors [23,24]. The primary objective of GMT is to assist patients with recognizing and reducing automatic responding during goal-directed behaviors and to promote the reinstatement of executive control. This is achieved through nine weekly two-hour sessions, including instructional material, interactive tasks, discussion of patients’ real-life deficits, and homework assignments. Each of the nine GMT sessions is detailed further in Table 1. Mindfulness skills are also incorporated for the purpose of learning how to bring one’s mind to the present to monitor ongoing behavior, goal states, and the correspondence between them. The program also incorporates real-life examples provided by the group facilitator and the participants to illustrate goal attainment failures and successes, as well as in-session practice on complex tasks that mimic real-life tasks that are problematic for individuals with executive function deficits (such as planning and attention).

In the present study, participants who attended at least one session of GMT, but terminated treatment before completing the majority (55% or 5/9) of the GMT sessions, were considered “drop-outs.” This value was determined by the expert opinion of clinicians experienced with the GMT protocol and has been applied in previous investigations (e.g., [30]). In the present study, one participant was counted as a dropout.

#### 2.3.2. WLC

Individuals randomized to this group were required to wait nine weeks, in addition to a three-month follow-up period after which they were invited to commence GMT.

### 2.4. Measures and Materials

#### 2.4.1. Clinical Interviews

MINI 6.0 or 7.0 [30]: The MINI is a brief, semi-structured, clinician administered interview assessing psychiatric disorders including mood and anxiety disorders according to the DSM-5 [2] and has high inter-rater reliability in psychiatric outpatients (κ > 0.75 for all disorders) [30].

CAPS-5 [33]: The CAPS-5 is a semi-structured interview assessing PTSD diagnostic criteria and symptom severity including frequency and intensity. The CAPS-5 has demonstrated high inter-rater reliability (κ = 0.78 to 1.00) and test-retest reliability (κ = 0.83), as well as convergent validity (r = 0.66 to 0.83) and discriminant validity (r = 0.02 to 0.54) in military veterans [33].

#### 2.4.2. Symptom Measures

Beck Anxiety Inventory (BAI) [34]: The BAI is a 21-item, self-report questionnaire that assesses anxiety symptoms over the past 30 days. The items consist of common symptoms of anxiety, such as numbness, tingling, sweating and fearing the worst. The BAI has demonstrated good internal consistency (α = 0.92 to 0.94), test-retest reliability (r = 0.67) and validity in psychiatric outpatients [34,35].

Beck Depression Inventory (BDI-II) [36]: The BDI-II is a 21-item, self-report questionnaire that assesses DSM-IV-TR defined symptoms of depression over the past 30 days. Symptoms assessed include hopelessness and irritability, feelings of guilt or feelings of being punished, as well as physical symptoms such as fatigue, weight loss, and lack of interest in sex. The BDI-II has demonstrated high internal consistency (α = 0.92), test-retest reliability (r = 0.93) and validity in psychiatric outpatients [37,38].

#### 2.4.3. Subjective Cognition

Cognitive Failures Questionnaire (CFQ) [39]: The CFQ is a 25-item self-report questionnaire that captures daily errors in distractibility, blunders, names, and memory, and has good test-retest reliability (α = 0.71) [40].

#### 2.4.4. Neuropsychological Assessment

Attention and response inhibition were tested using the Conners’ Continuous Performance Test-II (CPT-II) [41] in which individuals maintain their focus to respond to targets and inhibit response to non-targets. Processing speed was assessed using the Trail Making Test Part A (TMT-A) [42] and the Wechsler Adult Intelligence Scale-IV (WAIS-IV) Coding Subtest [43]. Processing speed and response inhibition were assessed using the Trail Making Test Part B (TMT-B) [42] and the Stroop Color and Word Test [44] in which color and word reading measured processing speed and the interference trial measured response inhibition. The Wisconsin Card Sorting Task (WCST) [45] was used to assess several executive function and related abilities, such as the ability to form and shift concepts. Finally, verbal memory was assessed using The California Verbal Learning Test-II (CVLT-II) [46], a word list learning task which provides raw and z-scores for immediate and delayed memory performance, interference learning and recognition. Normed scores were used for all analyses.

### 2.5. Data Analysis

Only 8.7% of data points were missing, with more missing data tending to be found among variables at the three-month follow-up appointment. The maximum number of data points missing from any variable was 26.7%. The majority (55%) of variables had between 0–3.3% of data points missing. Missing data were handled with expectation–maximization.

Repeated measures 2 (Group) × 3 (Time) ANOVAs were used for all outcome variables with estimates of partial eta-squared for effect size (interpreted conservatively as small = 0.01, medium = 0.10, and large = 0.25). Due to the exploratory pilot nature of this investigation, with emphasis on effect size rather than on statistical significance alone, all results were assessed with multivariate simple main effects regardless of significance of the overall ANOVA given that it was expected that this sample would be underpowered to reach significance in all cases. Multiple linear regression analyses were used for correlations between baseline PTSD severity and change in outcome variables. We used an alpha level of 0.05 for all statistical tests and all analyses were conducted using IBM SPSS version 26, IBM Corp.

## 3. Results

The mean age of participants was 49.41 (SD = 10.46) and 25% were male. The average level of education was 15.10 years (SD = 2.45) indicating completion of, at minimum, some post-secondary education among the majority of the sample. In total, 80% of participants in the GMT group and 93% of participants in the WL group self-reported being prescribed an anti-depressant at baseline (e.g., SSRI, SNRI, or Tricyclic). See Table 2 for further information regarding the medication status of participants. The mean BDI-II score at baseline was 29.72 (SD = 11.90) suggesting a level of moderate-to-severe depression in this sample. The mean BAI at baseline was 24.98 (SD = 14.47) indicating a moderate level of anxiety in this sample. The mean CAPS-5 score among trauma-exposed individuals was 19.10 (SD = 15.44). Intelligence quotient (IQ) was estimated using the Wechsler Abbreviated Scale of Intelligence, 2nd Edition [47], using one subscale from the verbal comprehension index (vocabulary) and one perceptual reasoning index (matrix reasoning). The mean IQ score of the sample was 105.02 (SD = 12.32), indicating average intelligence of the sample. See Table 2 for participant demographic and clinical characteristics by group.

### 3.1. Neuropsychological Assessment

Means and standard deviations as well as simple main effects results for all variables are presented in Table 2 for reference, and significant results are detailed below. A set of independent samples t-tests between variables indicated that there were no significant baseline differences between the two groups.

#### 3.1.1. Attention/Concentration and Processing Speed

A significant main effect of time (F(1,27) = 3.63, *p* = 0.040, η^2^_P_ = 0.168) was observed for the CPT-II d’ (an individual’s ability to differentiate targets from non-targets) with pairwise comparisons revealing that the GMT group improved significantly from time 1 to time 3 (*p* = 0.042) while the WLC group did not (*p* > 0.05). There was also a significant main effect of time for WAIS-IV Coding (F(1,27) = 5.89, *p* = 0.022, η^2^_P_ = 0.185) with pairwise comparisons revealing that the GMT group improved significantly from pre- to post-treatment (*p* = 0.044, η^2^_P_ = 0.146) while the WLC group did not (*p* = 0.368, η^2^_P_ = −0.63).

#### 3.1.2. Response Inhibition

A significant main effect of time was also observed for CPT-II commission errors (F(1,27) = 6.24, *p* = 0.019, η^2^_P_ = 0.189). Pairwise comparisons revealed that the GMT group improved significantly from pre- to post-treatment (*p* = 0.014, η^2^_P_ = 0.280) while the WLC group did not (*p* = 0.535, η^2^_P_ = 0.045). There was a significant main effect of time for the Stroop Color–Word trial (F(1,27) = 5.91, *p* = 0.022, η^2^_P_ = 0.167) as well as a near-significant interaction effect for the Stroop Interference score (F(1,27) = 4.03, *p* = 0.054, η^2^_P_ = 0.138). Here, pairwise comparisons showed that the GMT group improved significantly over treatment for the Color–Word trial (*p* = 0.018, η^2^_P_ = 0.295) and for the Interference score (*p* = 0.002, η^2^_P_ = 0.436) while the WLC group did not (*p* = 0.553, η^2^_P_ = 0.046 and *p* = 0.922, η^2^_P_ = 0.006).

#### 3.1.3. Self-Report Questionnaires

There was a near-significant interaction effect for CFQ score (F(1,27) = 4.12, *p* = 0.052, η^2^_P_ = 0.132), where pairwise comparisons revealed that the GMT group improved significantly from pre- to post-treatment (*p* = 0.034, η^2^_P_ = 0.228) while the WLC group did not (*p* = 0.930, η^2^_P_ = 0.005). There was also a significant main effect of time (*p* =0.042, η^2^_P_ = 0.144) for the BAI with pairwise comparisons indicating that the GMT group improved from time 1 to time 2 (*p* = 0.046) while the WLC group did not (*p* = 0.353).

#### 3.1.4. PTSD Severity

Multiple linear regression analyses were conducted with baseline CAPS-5 severity scores and changes in improved neuropsychological test scores and self-report questionnaires. These analyses were limited to participants in the GMT group that met criterion A for PTSD diagnosis: exposure to actual or threatened death, serious injury, or sexual violence (APA, 2013). Baseline symptom severity was negatively correlated with change in WAIS-IV coding (F(1,8) = 5.42, *p* = 0.045, β = −0.899), but there was no significant correlation with change in CPT-II d’(F(1,8) = 1.78, *p* = 0.231, β = −0.418). Change in response inhibition was not significantly correlated with baseline symptom severity for CPT-II commission errors (F(1,8) = 0.436, *p* = 0.528, β = −0.182), the Stroop Color–Word trial (F(1,8) = 0.09, *p* = 0.776, β = −0.048), or the Stroop Interference score (F(1,8) = 0.87, *p* = 0.378, β = −0.129). Change in the CFQ was also not significantly correlated with baseline symptom severity (F(1,8) = 0.206, *p* = 0.662, β = −0.158).

## 4. Discussion

The aim of the present study was to conduct an exploratory pilot investigation of the implementation of GMT in a sample of individuals with MDD, with an additional objective being to identify variables of interest for future large-scale investigations (i.e., those variables that demonstrate improvement in this pilot study). The secondary aim of the study was to explore how co-morbid PTSD symptom severity among trauma-exposed individuals with MDD may affect response to treatment. The results of this small pilot trial point towards focused and specific gains in neurocognitive performance on tasks related to attention, concentration and response inhibition following GMT administration. Specifically, improvements were observed for commission errors as well as detectability (d’) on the CPT-II, WAIS-IV Coding, and the Color–Word and Interference scores from the Stroop Task, relative to the WLC reference group. Effect sizes ranged from moderate (η^2^_P_ = 0.167) to large (η^2^_P_ = 0.436) highlighting a potentially strong treatment effect on these variables. Finally, there was also a significant improvement in ratings of subjective cognition in the GMT group. Importantly, all comparisons were significant between the pre- and post-treatment or pre-treatment and follow-up time points, indicating that these gains were made either exclusively during treatment or across the entire study window, and that no significant loss of gains was observed between post-treatment and the three-month follow-up assessment. Notably, among trauma-exposed individuals, heighted symptoms of PTSD were associated with diminished improvements on a measure of processing speed (WAIS-IV Coding), suggesting that co-morbid symptoms of PTSD may reduce response to treatment on this key index of cognitive performance. It is possible that the cumulative effects of PTSD and MDD on cognition [6,7] may account for the attenuated effect of treatment on processing speed. However, it is notable that comorbid PTSD symptoms did not attenuate the level of improvement on other variables assessed in this study (e.g., response inhibition, sustained attention, subjective cognitive difficulties). These findings are encouraging, suggesting that GMT may be equally effective in ameliorating cognitive difficulties among individuals with MDD with and without comorbid PTSD.

Critically, improvements in neuropsychological performance were observed on variables associated classically with cognitive impairment in MDD. GMT is designed to hone skills related to the brain’s sustained attention system, regulated by regions including the dorsolateral prefrontal cortex, posterior parietal and thalamic regions [24,48]. These regions may underscore important aspects of cognitive and behavioral therapies by improving concentration and attention in session, and by improving memory of content learned in session. Treatments for depression, including both antidepressants and cognitive behavioral therapies, have a positive impact on cognitive symptoms [11], but the pivot of focus toward cognitive remediation strategies may highlight the opposite end of the two-tailed hypothesis, that improving cognitive function may act to improve response to treatment. Indeed, particularly in relatively high-functioning samples, cognitive remediation might best be applied in MDD as an adjunctive treatment. Accordingly, future investigations of GMT may benefit from tailoring to the specific needs of the population (e.g., military members, public safety personnel and civilians) by incorporating relevant examples and in-session content. The reduced response to treatment of reductions in processing speed observed among individuals with more severe symptoms of PTSD also points to potential benefits for treatment approaches that address both disorders. A phased approach to treating comorbid MDD and PTSD with psychotherapy, for example, has shown high effectiveness in reducing symptoms [49].

Visual inspection of means and SDs in Table 2, as well as observations of effect size, suggest our sample may simply have been underpowered to reach statistical significance in some cases. Moreover, previous research suggests that improvements in cognitive function may reduce depressive symptoms [50]. Although not significant in our small sample, visual inspection of Table 2 reveals BDI-II scores fell from the low-severe range over treatment to the moderate range at post-treatment; these symptom scores remained relatively constant among waitlist controls. Further exploration of the impact of GMT on depressive symptoms is warranted.

The sample, overall, was relatively high functioning. In most cases, normed scores for both groups tended to be clustered near the average range with the majority of T-Scores falling between 45 and 55, and most of the improvements observed here moved scores from the low average to average range, or simply remained in the average range. Overall, however, this sample did not present with pronounced cognitive deficits beyond the low average range. Some variables, such as the WCST total correct, may have been impacted by ceiling effects. These observations underscore the importance of the large gains in subjective cognition observed here, particularly given that a nine-week program might not be of sufficient length or intensity to yield improvement in performance in cognitive domains that are very subtly impaired. In general, in disorders such as MDD and PTSD, where cognitive effects may be subtle (i.e., in the low average or mildly impaired range), it is critical that neuropsychological tests be of sufficient difficulty to capture subtle cognitive impairments, and not fall prey to ceiling effects.

Limitations from this pilot study can be used to inform future investigations. As stated, this sample may have lacked sufficient power to detect subtle performance deficits or small gains in performance. This is a common limitation of pilot investigations. Moreover, there is potential for Type I error in this study given the large number of comparisons performed, but this was deemed acceptable given the exploratory nature of the study. Additionally, we were unable to control for the effects of participation in other treatments for MDD (e.g., psychotherapy, medication); thus it is possible that improvements reported in this study may have been associated with participation in other treatments. However, this is unlikely as, as noted above, cognitive impairment has been reported to remain following remission of MDD [8]. There were a significant number of dropouts in this study. However, the majority of dropouts occurred prior to participation in GMT (e.g., after screening, but before initial testing, or after initial testing, but before the GMT group). One individual dropped out after the first GMT session. Although the reasons for drop-out was not recorded, it is possible that individuals dropped out due to the burden of participating in the assessment or the need to commit to a 9-week group treatment. Future studies may mitigate the impact of testing by selecting fewer measures based on previous findings, focused on core cognitive domains that are expected to improve following GMT. Notably, the dropout rate in our study is consistent with high dropout rates found across psychological interventions for depression [51], where it is possible that the symptoms of depression (e.g., anhedonia, low motivation) may interfere with initiation or completion of treatment. A major limitation of the study is that it did not explore the interaction of cognition and emotion through assessment of emotional regulation among individuals with MDD and the use of cognitive tasks that may be perturbed by emotion (e.g., the emotional Stroop). Strengths of the study included good completion of both post-treatment and three-month follow-up assessments, with relatively low missing data overall.

## 5. Conclusions

On balance, the results of this pilot study, although tempered by the small sample size and other limitations discussed above, are in line with previous investigations of GMT in various clinical populations in that we observed focused gains in some standardized variables, as well as improvements in subjective cognition. The burgeoning focus on non-traditional facets of MDD symptom presentation, such as alterations in cognitive performance (an important but often overlooked moderator of functional outcomes) represents a vital shift toward remediation of factors that may diminish response to pharmacological and non-pharmacological treatment. Improvements in cognitive performance would be expected to have downstream impacts on work and psychosocial functioning. Critically, treatment gains were observed primarily in cognitive subdomains that are affected in depressed samples and are targeted for improvement by GMT; thus revealing encouraging specificity of this intervention to individuals with MDD. PTSD symptoms, which are frequently comorbid in MDD [3], were associated with reduced response to treatment of alterations in processing speed, suggesting strongly the need to study further the impact of PTSD and trauma exposure on cognitive functioning in MDD and in treatment response. Subsequent studies of the use of GMT in neuropsychiatric conditions should include larger samples and an active control group to determine the specific effects of GMT relative to other treatment options.

## Figures and Tables

**Table 1:** Overview of Goal Management Training Protocol.

GMT Session	Description
Session 1: The Absent Mind, the Present Mind	Introduce the concept of absentmindedness and normalize the experience. Explain present-mindedness using mindfulness techniques.
Session 2: Absentminded Slip-Ups	Introduce construct of absentminded slips with examples, and discuss emotional and practical consequences. Introduce the “Body Scan” mindfulness exercise.
Session 3: The Automatic Pilot	Describe “automatic pilot” as being a habitual mechanism which can lead to inappropriate responses or actions if not monitored. Introduce the “Breathing Exercise” mindfulness technique.
Session 4: Stop the Automatic Pilot	Participants are introduced to the “STOP!” technique as a method of bringing one’s attention to the present to monitor current behavior. The short “Breath Focus” mindfulness exercise is described.
Session 5: The Mental Blackboard	The construct of working memory as a “mental blackboard,” which can be erased or over saturated with information, is explained. Participants are taught to check “the mental blackboard” to keep current goals at the forefront of memory. Introduce how to incorporate present-mindedness (specifically the “Breath Focus”) into behavior monitoring and executing difficult tasks as a method for increasing accuracy and memory.
Session 6: State Your Goal	Describe how goals can become entangled when attempting to multi-task. Introduce the concept of stating one’s goal as a way to aid encoding and recall of that goal.
Session 7: Making Decisions	Introduce the concept of conflicting goals and detail strategies for how to make decisions. Review methods for keeping track of complex goals using to-do lists.
Session 8: Splitting Tasks into Subtasks	Practice completing tasks that are too complex to rely on working memory only, and detail strategies for how to divide large goals into a series of smaller, more manageable subgoals.
Session 9: STOP!	Review the material covered across previous sessions and underscore the importance of goal monitoring (the “STOP!” technique).

Table reproduced from [30].

**Table 2:** Means and standard deviations for participant demographics and outcome variables for baseline, post-treatment and three-month follow-up times, with results of multivariate simple effects of time and pairwise comparison between timepoints.

**Variable**	**Group**	**Mean (SD)**			
Age	GMT	52.0 (8.77)			
	WLC	46.64 (11.69)			
Education	GMT	14.80 (2.01)			
	WLC	15.43 (2.90)			
**Medication**	**Group**	**%**			
Anti-depressant (e.g., SSRI, SNIR, Tricyclic)	GMT	80.00			
	WL	92.86			
Atypical Antipsychotic	GMT	20.00			
	WL	35.71			
Benzodiazepine	GMT	40.00			
	WL	28.57			
Anti-convulsant	GMT	20.00			
	WL	7.14			
Lithium	GMT	13.33			
	WL	7.14			
**Variable**	**Group**	**Time 1** **Mean (SD)**	**Time 2** **Mean (SD)**	**Time 3** **Mean (SD)**	**Pairwise Comparisons**
BAI Total Score	GMT	23.13 (12.54)	19,47 (11.96)	21.45 (13.17)	↓ T1 to T2 *
	WLC	27.00 (16.06)	27.50 (15.97)	25.71 (14.54)	*n.s.*
BDI Total Score	GMT	29.46 (11.33)	25.60 (13.73)	25.20 (11.90)	*n.s.*
	WLC	30.07 (12.42)	28.64 (14.28)	31.93 (12.04)	*n.s.*
CFQ Total Score	GMT	58.43 (13.09)	51.85 (16.82)	51.12 (13.92)	↓ T1 to T2 **
	WLC	59.64 (12.86)	60.79 (11.46)	60.07 (10.77)	*n.s.*
CVLT Trial 1–5 T-Score	GMT	52.27 (8.23)	59.00 (9.50)	57.50 (8.15)	*n.s.*
	WLC	53.79 (12.09)	56.50 (11.84)	57.21 (11.99)	*n.s.*
CVLT Short Delay Free Recall Z-Score	GMT	0.03 (0.72)	0.53 (0.94)	0.67 (0.81)	*n.s.*
	WLC	0.25 (1.24)	0.61 (1.11)	0.75 (1.31)	*n.s.*
CVLT Long Delay Free Recall Z-Score	GMT	−0.02 (0.62)	0.41 (0.85)	0.38 (0.88)	*n.s.*
	WLC	0.32 (1.28)	0.43 (1.22)	0.39 (1.36)	*n.s.*
CVLT Total Repetitions Z-Score	GMT	0.27 (1.19)	0.17 (1.04)	0.21 (0.89)	*n.s.*
	WLC	0.00 (0.76)	−0.11 (0.98)	−0.04 (1.18)	*n.s.*
CVLT Total Intrusions Z-Score	GMT	−0.20 (0.82)	−0.20 (0.89)	−0.21 (0.94)	*n.s.*
	WLC	−0.11 (0.63)	0.14 (0.89)	0.18 (1.19)	*n.s.*
Stroop Word T-Score	GMT	40.93 (7.79)	39.07 (7.41)	38.73 (8.51)	*n.s.*
	WLC	42.00 (10.96)	43.79 (12.27)	44.29 (12.74)	*n.s.*
Stroop Color T-Score	GMT	40.43 (5.98)	40.43 (8.93)	39.81 (8.76)	*n.s.*
	WLC	40.07 (8.06)	39.21 (10.05)	40.43 (9.77)	*n.s.*
Stroop Interference T-Score	GMT	48.29 (7.55)	52.36 (6.59)	49.84 (6.96)	↑ T1 to T2 *
	WLC	46.86 (9.41)	48.50 (7.36)	48.00 (8.77)	*n.s.*
Stroop Color-Word T-Score	GMT	46.32 (9.34)	51.00 (9.69)	50.23 (9.62)	↑ T1 to T2 ***
	WLC	51.21 (5.77)	51.29 (7.85)	51.65 (4.75)	*n.s.*
WASI Coding Scaled Score	GMT	10.50 (2.24)	11.07 (2.06)	11.09 (2.39)	↑ T1 to T2 **
	WLC	9.86 (3.09)	10.21 (3.31)	10.36 (3.59)	*n.s.*
TMT-A T-Score	GMT	49.79 (8.35)	52.00 (12.17)	52.63 (13.58)	*n.s.*
	WLC	47.00 (12.86)	49.50 (12.82)	52.64 (9.38)	↑ T1 to T3 *
TMT-B T-Score	GMT	50.29 (10.0)	51.36 (8.22)	52.18 (15.13)	*n.s.*
	WLC	48.57 (11.69)	52.71 (14.31)	53.29 (13.47)	↑ T1 to T3 *
WCST Total Correct T-Score	GMT	67.23 (11.20)	71.38 (11.33)	66.68 (6.14)	*n.s.*
	WLC	67.71 (9.24)	72.36 (10.76)	72.36 (13.12)	*n.s.*
WCST Total Errors T-Score	GMT	49.92 (9.89)	51.08 (7.85)	50.30 (11.07)	*n.s.*
	WLC	47.00 (11.34)	47.93 (8.90)	47.79 (8.95)	*n.s.*
WCST Perseverative Errors T-Score	GMT	50.61 (7.68)	51.69 (8.46)	51.80 (8.90)	*n.s.*
	WLC	47.93 (10.28)	48.86 (7.28)	48.64 (9.190	*n.s.*
WCST Non-Perseverative Errors T-Score	GMT	49.38 (11.64)	49.69 (7.36)	47.90 (12.13)	*n.s.*
	WLC	46.71 (10.99)	46.21 (9.36)	46.29 (8.41)	*n.s.*
CPT Omission Error T-Score	GMT	57.99 (12.57)	51.42 (14.66)	51.26 (12.38)	*n.s.*
	WLC	51.97 (12.46)	50.83 (6.95)	54.92 (14.13)	*n.s.*
CPT Commission Errors	GMT	52.63 (9.44)	45.93 (7.91)	46.11 (6.93)	↓ T1 to T2 *
	WLC	53.31 (13.14)	50.84 (12.04)	50.45 (12.63)	*n.s.*
CPT Hit Reaction Time T-Score	GMT	55.10 (11.41)	56.49 (8.63)	53.70 (8.01)	*n.s.*
	WLC	57.83 (11.80)	55.27 (11.72)	58.20 (12.43)	*n.s.*
CPT Variability T-Score	GMT	54.71 (9.46)	51.24 (11.48)	54.50 (11.53)	*n.s.*
	WLC	56.59 (15.06)	53.28 (13.11)	52.98 (16.11)	*n.s.*
CPT d’ T-Score	GMT	49.40 (10.06)	46.09 (9.65)	45.89 (9.49)	↑ T1 to T3 *
	WLC	51.14 (13.96)	50.03 (10.37)	47.62 (10.72)	*n.s.*

CPT T-Scores are interpreted as higher = worse performance. Partial eta-squared interpreted approximately as small = 0.01; medium = 0.10 and large = 0.25. Results reported in this table are for the simple main effects, not the overall ANOVAs. *n.s.* = No significant differences between any time points. *Legend.* BAI = Beck Anxiety Inventory; BDI = Beck Depression Inventory; CFQ = Cognitive Failures Questionnaire; CPT = Conners’ Continuous Performance Test 2nd Edition; CVLT = California Verbal Learning Test-Second Edition; Stroop = Golden Stroop Task; TMT = Trail-Making Task-A/B; WASI = Wechsler Adult Scale of Intelligence; WCST = Wisconsin Card Sorting Test. * Pairwise comparison *p* < 0.05 ** Pairwise comparison *p* < 0.01 *** Pairwise comparison *p* < 0.001 *n.s.* Pairwise comparison nonsignificant. ↓ decreased; ↑ increased.

## Data Availability

Transfer of data outside the housing institution is currently not supported by the institution’s ethics policy.
